# Assessment of a new point-of-care system for detection of prostate specific antigen

**DOI:** 10.1186/s12894-016-0119-9

**Published:** 2016-01-19

**Authors:** Steffen Rausch, Joerg Hennenlotter, Josef Wiesenreiter, Andrea Hohneder, Julian Heinkele, Christian Schwentner, Arnulf Stenzl, Tilman Todenhöfer

**Affiliations:** Department of Urology, Eberhard-Karls-University Tuebingen, Hoppe-Seyler-Str. 3, 72076 Tuebingen, Germany; Vancouver Prostate Centre, University of British Columbia, 2660 Oak Street, Vancouver, V6H 3Z6 Canada

**Keywords:** Point-of-care, Prostate cancer, Prostate specific antigen

## Abstract

**Background:**

Measurement of the prostate specific antigen (PSA) remains an important tool in prostate cancer (PC) diagnosis. Due to limited availability of laboratory devices in an outpatient setting, compact and easy-to-handle point-of-care (POC) systems are desirable. Recently, a chip for PSA measurement on the concile® Ω100 POC reader platform was introduced. To investigate the clinical applicability, we evaluated the system in a consecutive cohort of patients undergoing PSA measurement in our outpatient clinic.

**Methods:**

Between 07/2014 and 01/2015, PSA was analyzed in a total of 198 patients by the POC reader system and in parallel by an Immulite 2000® and Centaur® standard laboratory system, respectively. By standard (Immulite®) measurement, 67 (34,2 %) had PSA > 4 ng/ml and 131 (65,8 %) had PSA ≤ 4 ng/ml. Results were correlated by linear regression analyses for all patients and within PSA subgroups. For patients with available prostate histology after PSA measurement (*n* = 68), receiver-operating characteristic curves were created and area under the curve (AUC), sensitivity and specificity for the prediction of PC at best cut-off value were calculated.

**Results:**

The coefficients of determination (r^2^) for the POC device compared to laboratory testing were 0.72 (Immulite®) and 0.63 (Centaur®), respectively (both *p* < 0.0001). In the PSA range of ≤4 ng/ml, the observed correlations were 0.75 and 0.70, respectively. For the POC test system, AUC for detection of PC was calculated with 0.745 while the standard laboratory tests showed 0.778 (Immulite®) and 0.771 (Centaur®). At best cut-off of 3.64 ng/ml, PSA analysis by the POC system showed a sensitivity of 85.7 % and a specificity of 66.7 %.

**Conclusions:**

The POC system obtained good concordance to elaborate laboratory measurement. In a screening scenario, the system provides quick and reliable PSA measurement, especially in the PSA range up to 4 ng/ml.

## Background

Since its discovery in 1979 by Wang et al. [1] prostate specific antigen (PSA) has become the gold standard biomarker for screening, diagnosis, and therapeutic monitoring of prostate cancer (PC), and a routine clinical parameter for the management of benign hyperplasia and inflammatory disorders of the prostate [2–7].

Despite country and health-system dependent approaches to prostate cancer screening and PSA testing, patients with clinical symptoms of urinary and prostatic disorders or the intention to undergo screening for prostate cancer are often primarily seen and tested for PSA in an outpatient setting. However, laboratory tests for PSA are usually performed in centralized institutions and therefore most often not available in proximity to the urologists’ or general practitioners’ office, resulting in a delayed information and putative psychological discomfort for patients [8].

Point-of-care (POC) test systems for a rapid and reliable testing at the practitioners’ office have been already introduced for several medical conditions. For the evaluation of diabetes or acute inflammation, POC systems measuring blood count, CRP or HbA1c have shown to provide reliable test results [9–12].

The present prospective study was performed in order to evaluate the reproducibility and clinical applicability of a novel quantitative POC PSA assay (CancerCheck® PSA, concile GmbH, Freiburg, Germany) using a portable device (concile® Ω100) in comparison to established standard routine laboratory PSA test systems.

## Methods

### Patient cohort

We prospectively included 200 patients, who underwent routine PSA measurement at the University hospital of Tuebingen. To cover a broad range of PSA values, patients were recruited based on PSA analysis using the Immulite® system, which was set as reference method. We evaluated 150, 25 and 25 patients with PSA values in the range of ≤4 ng/ml, 4–10 ng/ml and >10 ng/ml. Patients referred for PC screening, and patients designated to undergo surgery for PC or lower urinary tract symptoms due to benign prostatic hyperplasia were included. Patients with a history of other urogenital malignancy were excluded from the study.

Patient samples were prospectively analyzed with two elaborated test systems, Immulite 2000® (Siemens Healthcare Diagnostics, Deerfield, USA) and Centaur® (Siemens Healthcare Diagnostics), and the POC test system (CancerCheck® PSA, on the concile® Ω100 reader, concile GmbH, Freiburg, Germany) in parallel. Blood samples were collected one day prior to the analyses and stored at 2–8 °C to provide simultaneous application of the tests. All three tests were performed within a time-span of one hour.

Baseline patients’ characteristics (age, history of prior prostatic disease), subsequent interventions and clinical parameters after PSA-measurement (biopsy, surgery, histology) were recorded.

Written informed consent was obtained by all participants, and the study was approved by the local ethics committee of the University hospital Tübingen (No. 122/2012BO2).

### POC-Test system principle

The POC system consists of a portable device (concile® Ω100 reader, and a test cassette containing a one-step chromatographic sandwich immunoassay, which is analyzed by a charged-couple device (CCD).

**Fig. 1:**
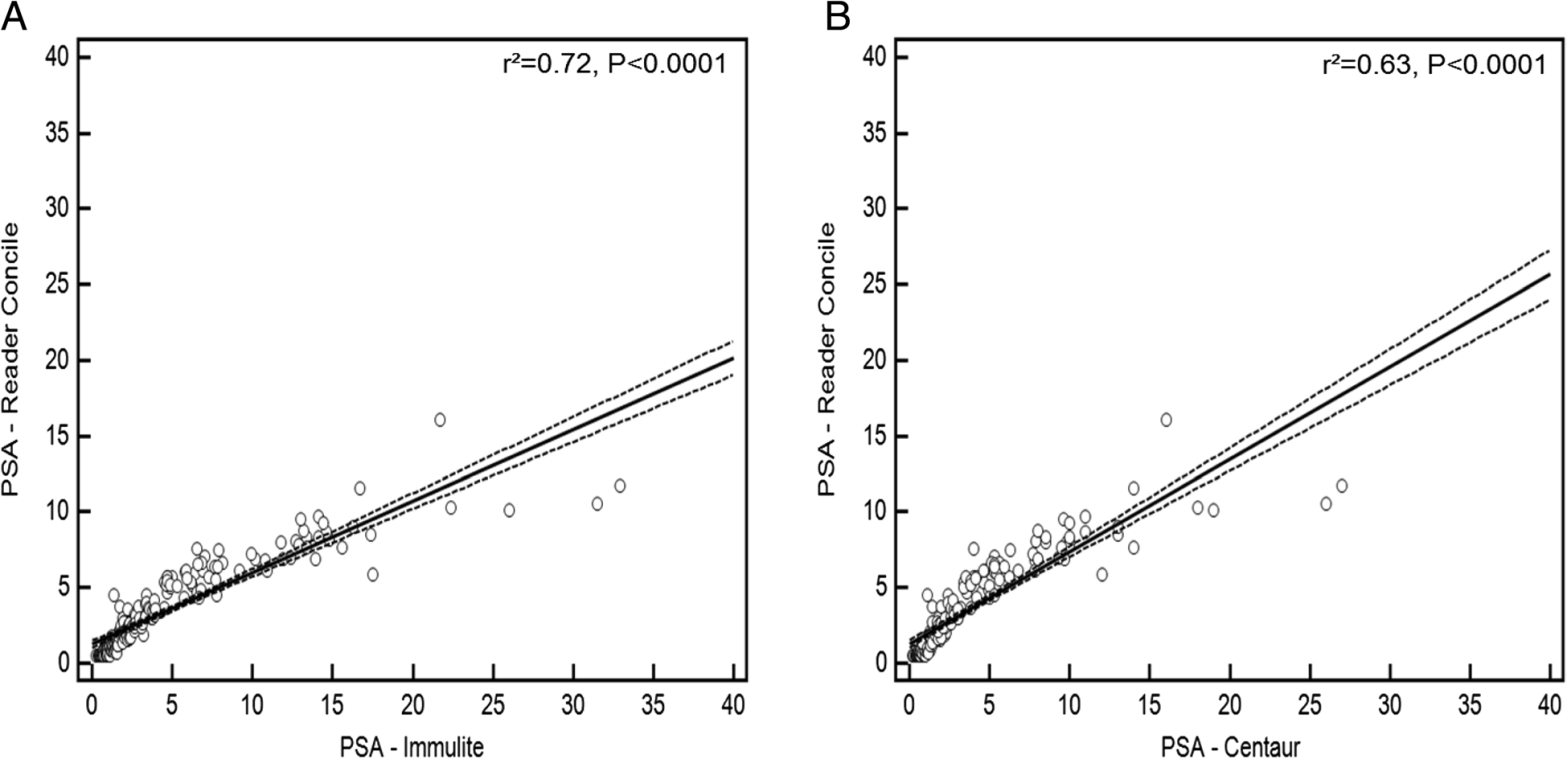
Illustration of concile® PSA measurement compared to PSA-Immulite®(**a**) and PSA-Centaur®(**b**). (for reasons of graphical illustration, two measurements in the high PSA range are not displayed.)

After addition of sample, PSA binds to a colloidal gold-labeled antibody. The resulting complex is captured by a specific antibody pre-coated at the test zone and forming a red test line. The concentration of PSA is measured using complimentary light and the intensity of the test line is measured by the CCD sensor. The volume of the signal is directly proportional to the concentration of PSA in the sample and can be measured by the concile® Ω100 reader with a pre-set calibration curve. The concentration of PSA is displayed as numeric result with one decimal place.

### Statistics

For data analyses, patients were classified into different categories of total PSA (tPSA) range ≤4, ≤10 ng/ml and >10 ng/ml according to the Immulite® measurement.

Linear regression analyses were performed to correlate individual test results given by the POC reader and Immulite® and Centaur® measurement in the whole group and in the three subgroups, respectively. In the subgroup of patients with available histology, receiver-operator-characteristics (ROC) analyses were performed to evaluate the diagnostic performance of the tests with respect to the correct identification of PC and the area-under-the-curve (AUC) was calculated. Youden’s index was used to identify the optimum cut-off for each test system. Comparison of ROC curves was performed according to the method of DeLong. Analyses were performed using commercial software (MedCalc, version 12.5, Ostend, Belgium). Statistical significance was defined as *p* < 0.05.

## Results

Samples of a total of 198 (99 %) patients were available for the analyses. Two patients were excluded from the collective due to insufficient sample preparation or extreme PSA values (>800 ng/ml). Median patient age was 66.15 years. Histological workup was performed in 68 patients (34.7 %). Of those, *n* = 36 (52.2 %) were diagnosed with PC. Patients’ characteristics are summarized in Table 1.

**Table 1:** Patients’ characteristics

Median patient age, years (Range)	66.15	(42.06–90.09)	
Patient groups according to Immulite® measurement		*n*=	%
	≤4 ng/ml	131	65.8 %
	>4 ng/ml	67	34.2 %
	≤10 ng/ml	168	84.8 %
	>10 ng/ml	30	15.2 %
Further diagnostic workup			
Histology available^a^	Yes	69	34.7 %
	No	130	65.3 %
Histologic evidence of prostate cancer	Yes	36	52.2 %
	No	30	43.5 %
		Median	95 % CI
PSA values	Immulite®	2.34	2.07 to 3.10
	Centaur®	1.90	1.70 to 2.33
	concile®	2.53	2.10 to 3.15

^a^from radical prostatectomy, TUR-P, or prostate biopsy

**Fig. 2:**
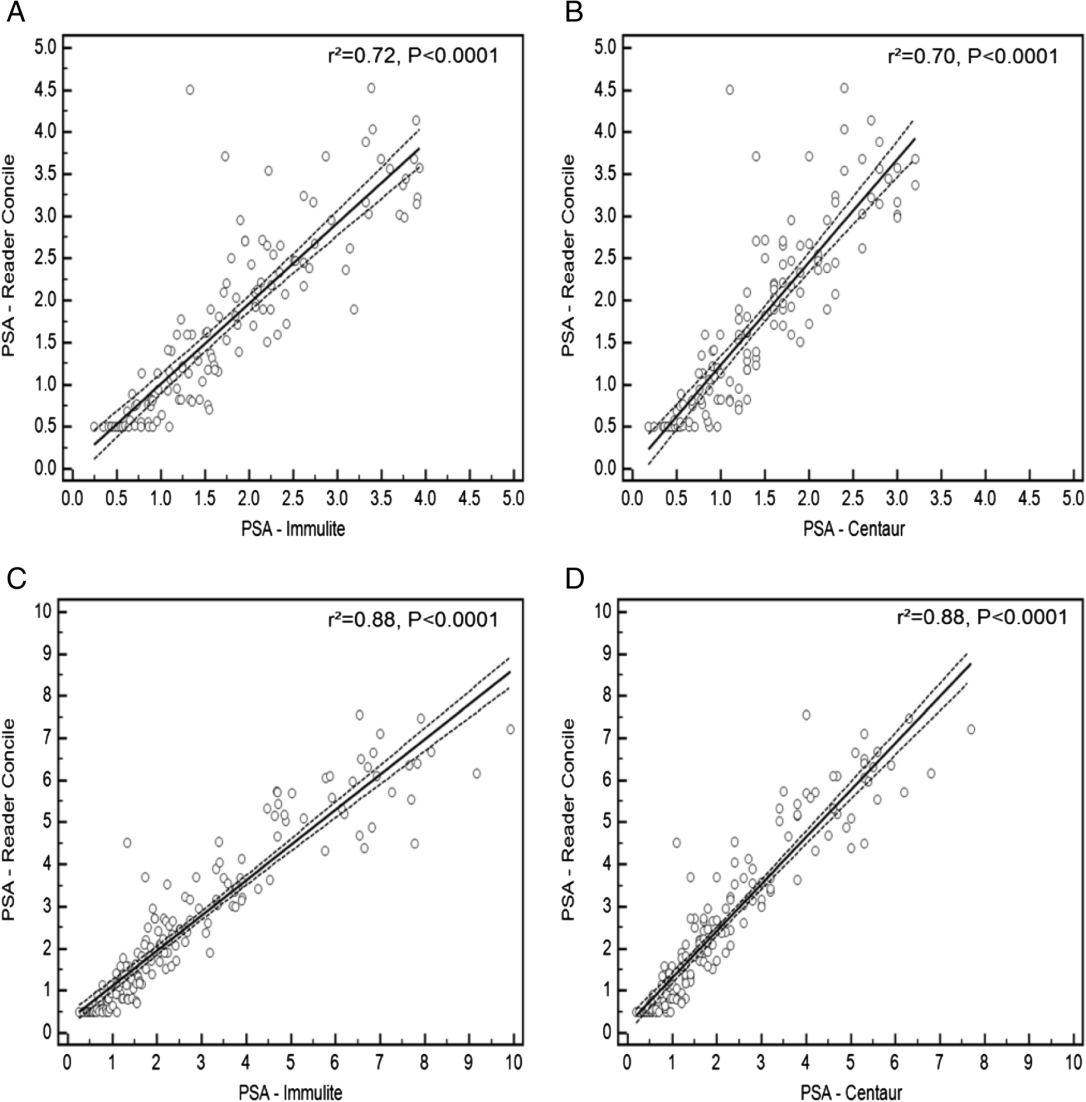
Linear regression analysis of concile® PSA measurement in comparison to Immulite® (**a**) and Centaur (**b**) values at PSA range ≤ 4 ng/ml and ≤ 10 ng/ml (**c**, **d**) (dashed lines = 95 % confidence interval)

**Fig. 3:**
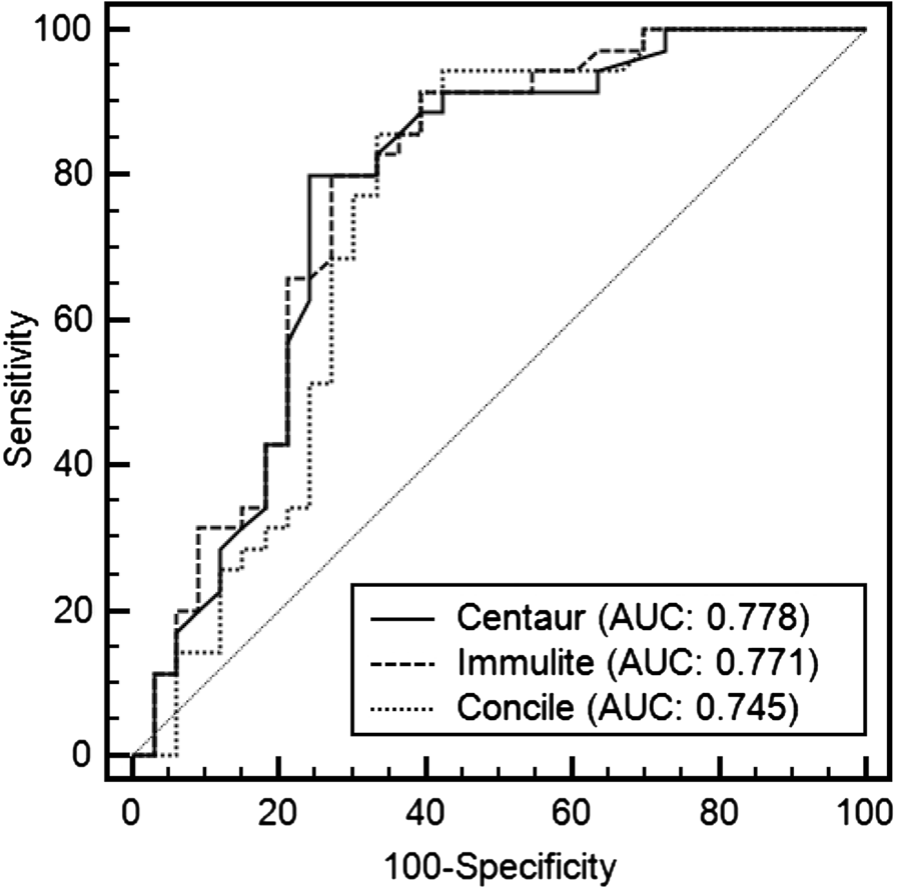
Comparison of receiver operator chararcteristic curves resulting from the three test systems

Figure 1 illustrates PSA values determined by POC concile® measurement compared to the Immulite® (A) and Centaur® test results (B). The correlation of the three systems in different PSA ranges is illustrated in Fig. 2. The coefficient of determination (r^2^) for the POC measurement compared to routine lab testing was 0.72 (Immulite®) and 0.63 (Centaur®), respectively. In the PSA ≤4 ng/ml subgroup, r^2^ were 0.75 (Immulite®) and 0.70 (Centaur®) (both *p* < 0.0001). For patients with PSA ≤10 ng/ml the coefficients of determination observed were both 0.88 (Immulite® and Centaur®), respectively. In the high PSA group of >10 ng/ml, r^2^ was 0.72 (Immulite®) and 0.70 (Centaur®) (Table 2). With regard to the correct prediction of prostate cancer, ROC analysis (Fig. 3) revealed an AUC of 0.745 (95 % CI: 0.625 to 0.843) for the POC test system, while the AUCs for the standard laboratory tests were calculated with 0.778 (95 % CI: 0.661 to 0.870) (Immulite®) and 0.771 (95 % CI: 0.653 to 0.864) (Centaur®). The comparison of ROC curves revealed no significant difference between POC and Centaur® measurement, while a significant difference of AUCs from POC and Immulite® analyses was noted (*P* = 0.03) (Table 3). From ROC analysis, optimal PSA cut-off values for the individual PSA test systems were determined. Sensitivity and specificity for the detection of prostate cancer and optimal PSA cut-off values for the respective test systems are shown in Table 4. At best cut-off (3.64 ng/ml), the POC system showed a sensitivity of 85.7 and a specificity of 66.7, respectively. Table 5 illustrates the association between PSA at cut-off 4 ng/ml and the presence of PC in the sub-cohort of patients with available histology. POC measurement showed the highest negative predictive value (81.5 %). No further false negative result was observed compared to Immulite®, however two patients (2.9 %) that were negatively tested in the Centaur® assay were correctly classified as PC patients by POC measurement.

**Table 2:** Results from linear regression analysis

	Collective (*n* = 198)		PSA ≤ 4 ng/ml (*n* = 131)		PSA ≤ 10 ng/ml (*n* = 161)		PSA > 10 ng/ml (*n* = 37)	
	r^2^	*P*=	r^2^	*P*=	r^2^	*P*=	r^2^	*P*=
Concile® vs. Immulite®	0.7181	<0.0001	0.7518	<0.0001	0.8775	<0.0001	0.7155	<0.0001
Concile® vs. Centaur®	0.6346	<0.0001	0.7037	<0.0001	0.8782	<0.0001	0.7031	<0.0001

**Table 3:** Comparison of receiver operator characteristic curves (*CI* confidence interval, *SE a* standard error, *PSA* prostate specific antigen)

	Difference between areas	Standard error	95 % CI	z statistic	Significance level
PSA Immulite® vs. PSA concile®	0.0333	0.0162	0.00165 to 0.0650	2062	*P* = 0.0392
PSA Centaur® vs. PSA concile®	0.026	0.0201	-0.0134 to 0.0654	1293	*P* = 0.1961

**Table 4:** Prostate specific antigen best cut-off, sensitivity and specificity from receiver operator characteristic analysis

	Best cut off	Sensitivity	95 % CI	Specificity	95 % CI
Immulite®	5.02	80.0	63.1–91.6	72.7	54.5–86.7
Centaur®	4.00	80.0	63.1–91.6	75.8	57.7–88.9
Concile®	3.64	85.7	69.7–95.2	66.7	48.2–82.0

**Table 5:** Performance of different standard and POC assays at PSA cut-off 4 ng/ml (*CI* confidence interval, *PC* prostate cancer, *PSA* prostate specific antigen, ^a^ from chi-square test)

	*PSA Immulite*®				*PSA Centaur*®				*Concile*®		
	No PC	PC			No PC	PC			No PC	PC	
PSA < 4	20	5	25 (36.8 %)	PSA < 4	25	7	32 (47.1 %)	PSA < 4	22	5	27 (39.7 %)
PSA ≥ 4	13	30	43 (63.2 %)	PSA ≥ 4	8	28	36 (52.9 %)	PSA ≥ 4	11	30	41 (60.3 %)
	33 (48.50 %)	35 (51.50 %)	68		33 (48.50 %)	35 (51.50 %)	68		33 (48.50 %)	35 (51.50 %)	68
Significance level^a^	P = 0.0002				P < 0.0001				P < 0.0001		
		95 % CI				95 % CI				95 % CI	
Sensitivity	85.71 %	69.74 to 95.19 %			80.00 %	63.06 to 91.56 %			85.71 %	69.74 to 95.19 %	
Specificity	60.61 %	42.14 to 77.09 %			75.76 %	57.74 to 88.91 %			66.67 %	48.17 to 82.04 %	
Positive predictive value	69.77 %	53.87 to 82.82 %			77.78 %	60.85 to 89.88 %			73.17 %	57.06 to 85.78 %	
Negative predictive value	80.00 %	59.30 to 93.17 %			78.12 %	60.03 to 90.72 %			81.48 %	61.92 to 93.70 %	

Within POC measurement, the rate of false positive subjects at cut-off PSA 4 ng/ml was 16.2 % (*n* = 11), while the rates of Centaur® and Immulite® were 11.8 (*n* = 8) and 19.1 % (*n* = 13). On the other hand, rates of patients with a PSA value >4 ng/ml in the Immulite® or Centaur® but <4 ng/ml in POC analysis were 1.0 % (*n* = 2, Immulite®) and 0 % (*n* = 0, Centaur®), respectively (Table 6).

**Table 6:** Comparison of different standard and POC assays at cut-off PSA 4 ng/ml

	PSA < 4 Concile®	PSA ≥ 4 Concile®			PSA < 4 Concile®	PSA ≥ 4 Concile®			PSA < 4 Centaur®	PSA ≥ 4 Centaur®	
PSA < 4 Centaur®	129	13	142 (71.7 %)	PSA < 4 Immulite®	127	4	131 (66.2 %)	PSA < 4 Immulite®	131	0	131 (66.2 %)
PSA ≥ 4 Centaur®	0	56	56 (28.3 %)	PSA ≥ 4 Immulite®	2	65	67 (33.8 %)	PSA ≥ 4 Immulite®	11	56	67 (33.8 %)
	129 (65.2 %)	69 (34.8 %)	198 (100.0 %)		129 (65.2 %)	69 (34.8 %)	198 (100.0 %)		142 (71.7 %)	56 (28.3 %)	198 (100.0 %)

## Discussion

In the present study, we detected a close correlation between a new POC test system and standard laboratory tests, as documented by a coefficient of determination of 0.72 for the overall patient population comparing concile® Ω100 reader and Immulite® measurement. In the clinically relevant PSA range of ≤4 ng/ml with regard to the prediction of a negative result in a PC screen scenario, the observed correlation was even higher, with r^2^ of 0.75.

Nevertheless, AUC analysis revealed a higher accuracy for the established standard assays, which has also been reported in earlier publications on POC PSA test systems [13].

However, in urologist’s daily practice it is well known, that even the established laboratory systems differ in their results. Therefore, the decision of clinicians whether a biopsy should be recommended or not is dependent on the PSA system used. Slev et al. analyzed the intermethod differences for six different laboratory PSA assays, including Immulite® and Centaur® and reported relative differences of more than 10 % at PSA of 4.0 ng/ml [14].

In this context it is noteworthy that a PSA-POC system may not provide meticulous correlation to all of the standard laboratory tests, it should however try to give the PSA value on a level that is located in an appropriate range compared to standard assays. A valid variable for determining this level is the comparison of individual system’s best cut- offs. With 3.64 ng/ml the POC system ranged in its level at an adequate best cut-off value.

A PSA value of 4 ng/ml is considered a common threshold for a biopsy decision. At a cut-off PSA value of 4 ng/ml, POC measurement outperformed Immulite® and Centaur® with regard to the negative predictive value, which underlines the effectiveness of POC measurement as a screening tool. POC test systems used at a general practitioners office could be used as pre-screening tests and avoid unnecessary referrals to urologists in cases of inconspicuous digital rectal examintation and low POC PSA values. Despite the fact, that PSA standard lab test results may in some cases be available within a few hours according to specific health system dependent or institutional conditions, the main rationale for the use of POC tests is the option to receive a test result within 20 min, which makes a discussion of the test result with the patient possible in the same session.

As a general rule, POC tests should not be applied as a diagnostic following radical prostatectomy, where ultrasensitive monitoring of PSA is recommended [15, 16]. In patients with POC PSA values in the range of 2.5 to 4.0 ng/ml [17] and beyond, POC measurement should be regarded as a pre-screening test and an immediate routine lab testing should follow. In addition, the confirmation of an elevated PSA after three weeks, as recommended by current PC treatment guidelines in cases of cancer suspicion, should not exclusively been performed by concile® Ω100 measurement [15]. Hence, POC measurement is subject to the same restrictions in men undergoing active surveillance for PC. However, the POC assay appears appropriate for the identification of patients with a low risk of prostate cancer in the PSA range of <2.5 ng/ml. The evaluation of other frequent prostatic diseases like benign hyperplasia, prostatitis, and follow-up studies for prostate cancer after radiotherapy or hormonal treatment may also be performed based on concile® PSA analysis. In PSA ranges >10 ng/ml, up to extreme PSA values, the diagnostic precision of POC measurement is impaired. Follow up studies in patients with extreme PSA values should therefore be performed with standard assays.

By using PSA POC measurements, the regularly observed delay between PSA sampling, receipt of test results, and the information and discussion with the patient may be overcome for a large subgroup of patients. In a prospective study of 188 patients, Wilkinson et al. observed that 89 % of patients receiving a rapid result would prefer to have this method again in order to facilitate the discussion regarding their future management. However, no significant differences between stress and anxiety of patients receiving PSA test results within 15 min after the test, and 1–4 days after PSA testing were detected [18].

Handling of the POC system was favorable. While other one-step POC test systems, usually based on lateral flow chromatographic immunoassays, could demonstrate practical feasibility and good correlation to routine PSA lab values [13, 19, 20], earlier studies with semi-quantitative strip tests for the evaluation of PSA in whole blood, failed to prove their clinical utility due to impaired test handling and interpretation [21].

## Conclusions

POC measurement with the herein evaluated system allows rapid quantitative analysis of PSA and may help to circumvent limitations of ambulatory PSA testing for patients and physicians in need of an immediate test result.

## Abbreviations

AUC: area under the curve
CCD: charged-couple device
PC: prostate cancer
POC: point-of-care
PSA: prostate specific antigen
ROC: receiver-operator characteristic
